# Pre-operative COVID-19 screening: a model to provide non-discretionary care for urologic patients

**DOI:** 10.1590/S1677-5538.IBJU.2020.0516

**Published:** 2020-12-20

**Authors:** Basil A. Ferenczi, Ron Ron Cheng, Adam Daily, Christian Kuhr, Kathleen Kobashi, John M. Corman

**Affiliations:** 1 Virginia Mason Medical Center Section of Urology SeattleWashington USA Virginia Mason Medical Center, Section of Urology, Seattle Washington, USA

## INTRODUCTION

COVID-19 in Washington State has led to unprecedented challenges within the Urologic community as physicians work to provide care that is safe for patients and staff. In order to conserve personal protective equipment (PPE) and to ensure hospital capacity for COVID-19 infected patients, Washington State Governor Jay Inslee directed that elective surgical procedures should be suspended on March 19, 2020 (1). However, non-elective Urologic care still needed to be provided.

Preventing transmission of COVID-19 has been of paramount concern during the pandemic. Procedural care is at particularly high risk with its associated aerosol-generating procedures: intubation and extubation (2). Patients cannot be screened for infection solely based on symptoms, as a significant number are asymptomatic (3, 4), and many carriers never develop symptoms(5). Providing fit-tested N95 masks to all procedural staff is not currently feasible given the international shortage of PPE (6).

Furthermore, pre-test probability of infection is difficult to estimate as our community's COVID-19 burden has not been established, and studies have demonstrated significant geographic variability within the United States (7, 8). Further complicating the picture are the wide variety of available testing modalities with a range of sensitivity, specificity, and negative and positive predictive values. The majority of these have been FDA approved under emergency use authorization (9).

The harm in suspending Urologic care to the community is significant. Increased surgical waiting time (SWT) for T3 renal masses has been associated with decreased overall survival (10), and a delay in bladder cancer treatment has been demonstrated to lead to worse prognosis and higher pathologic stage (11). Based on Organ Procurement and Transplantation Network Data as of May 1, 2020 there has been nearly a 50% decrease in the number of kidney transplants performed in mid-March compared to mid-April, impacting a pre-existing shortage in available organs (12). Delayed relief of ureteral obstruction is associated with long-term renal dysfunction (13). Finally, the psychological impact of a delay in surgical care cannot be underestimated, affecting patient anxiety level and general health perceptions (14).

In order to safely provide care for those who may be harmed by a treatment delay, on April 1st, 2020 Virginia Mason Medical Center committed to screen all patients prior to any surgical care. This implementation appears to be an effective measure to protect patients and staff, with no known COVID-19 cases in perioperative staff since the advent of screening. The primary objective of this study was to evaluate the impact of pre-operative COVID-19 screening on our ability to provide Urologic care. This was measured using Urologic surgical volume during an interval when discretionary surgery was suspended.

## MATERIALS AND METHODS

Testing: Effective April 1, 2020 all pre-procedural patients were tested for COVID-19. Testing occurred within 48 hours prior to the scheduled intervention or at the time of hospital admission. A nasopharyngeal swab specimen was collected and processed using the Abbott RealTime SARS-CoV-2 assay. Mid-turbinate testing was substituted for nasopharyngeal swabs on May 3, 2020 in accordance with expanded CDC sampling guidelines (15). Patients who screened positive for COVID-19 were rescheduled to a later date. If medically stable, they were discharged home, and rescheduled for surgery following two subsequent negative repeat screening tests. In emergent situations, patients were either screened with a rapid ePLEX SARS-CoV-2 test or their procedure was performed in a specially engineered negative air pressure “COVID pod,” utilizing Powered Air-Purifying Respirators or fitted N95 face masks and eye protection. PPE for patients who tested negative for COVID-19 included standard surgical masks and protective eye shields.

Stratification: All cases were triaged into one of five tiers: Emergent, Urgent, Planned Procedure level 1, Planned Procedure level 2, and Discretionary Procedure (Table-1). Proposed procedures were reviewed by an independent multidisciplinary committee to ensure that purely discretionary procedures (defined as a delay in performing the intervention would not result in harm to the patient) were not performed during the March 19 to May 18, 2020 prohibition period.

**Table 1:** Triage levels used to determine case urgency during the COVID-19 pandemic with corresponding urologic procedures (non-exhaustive list).

Triage Level	Example of Procedures
Emergent	Fournier's gangrene debridement, decompression for obstructive pyelonephritis
Urgent	Decompression of symptomatic nephrolithiasis, cystoscopic fulguration for active bleeding
Planned Procedure Level 1	Transurethral resection of high-grade bladder tumor
Planned Procedure Level 2	Radical prostatectomy for high-risk prostate cancer, deceased donor renal transplant
Discretionary	Inflatable penile prosthesis insertion, mid-urethral sling

### Data Collection and Analysis

Data regarding Urologic operative volume was collected retrospectively. Only procedural care based in the operating room was included in the analysis. Comparison of surgical volumes was performed between baseline [one year prior to the COVID-19 pandemic (March 19-May 6, 2019)], pre-intervention (March 19-March 31, 2020), and post-intervention (April 1-May 6, 2020) time periods. All statistical analyses were 2-sided, and significance was defined as p <0.05. Statistics were performed using STATA v13.0 (StataCorp, College Station TX).

## RESULTS

Screening: As an institution, 840 asymptomatic patients were screened from April 1, 2020-May 6, 2020. Three patients (4%) tested positive. A total of 126 urology cases were performed and a total of 118 urology patients were screened. None were positive.

Operative Volume: Baseline: March 19 through May 6, 2019, 295 Urologic surgeries were performed (6.0 surgeries/day (SD=4.5)). These cases were categorized as follows: 1.9 cases/day general urology (31.9%), 1.4 cases/day oncology (23.7%), 1.2 cases/day endourology (19.7%), 0.9 cases/day female pelvic medicine and reconstructive surgery (FPMRS) (14.6%), 0.5 cases/day transplant (8.1%) and 0.1 cases/day reconstruction (2.0%).

Prior to pre-surgical COVID-19 screening: March 19 through March 31, 2020, 21 cases were performed (1.6 surgeries/day (SD=1.9)). Of these cases, 0 were defined as emergent or urgent, 13 were defined as planned level 1, 8 were defined as Planned level 2, and 0 were defined as discretionary. These cases were categorized as follows: 0.2 cases/day general urology (14.3%), 0.5 cases/day oncology (28.6%), 0.6 cases/day endourology (38.1%), 0.3 cases/day FPMRS (19.0%), and 0 reconstruction and transplant.

After initiation of pre-surgical COVID-19 screening: April 1st through May 6, 2020, 126 Urologic surgeries were performed, (3.5 surgeries/day (SD=2.9)). Of these cases, 1 was defined as emergent, 12 were defined as urgent, 73 were defined as planned level 1, 39 were defined as Planned level 2, and 1 was defined as discretionary. These cases were categorized as follows: 0.9 cases/day general urology (24.6%), 1.2 cases/day oncology (34.9%), 1.0 case/day endourology (28.6%), 0.3 cases/day FPMRS (7.14%), 0.1 cases/day reconstruction (2.4%), and 0.1 cases/day transplant (2.4%).

The Urologic operative volume was significantly different between baseline and the pre-screening period (p=0.001), between baseline and the post-screening period (p=0.004), and between the pre-screening and post-screening period (p=0.036). There was no significant difference between age or gender distribution between any treatment period. There was a significant difference in the distribution of urology cases performed stratified by subspecialty between all three treatment periods (p=0.008).

## DISCUSSION

This study demonstrates the utility of pre-operative screening as a means to safely expand Urologic care during the COVID-19 pandemic. Following the institution of this policy, the Virginia Mason Urologic service cared for more than twice the number of patients and mitigated the harm to our community caused by prolonged SWT. Compared to baseline, there was a trend towards higher proportion of oncologic and endourologic cases and fewer transplant cases performed in both pre-screening and post-screening eras. Of interest, the proportion of FPMRS initially increased and then declined precipitously.

The inability to access timely Urologic care has an adverse impact on society. An absence of Urologic care has been shown to increase disability-adjusted life years in regions without access to care (16). Surgical waiting time is well established as a risk of Urologic disease progression. Fahmy et al. performed a meta-analysis to evaluate the effect of delay in cystectomy on patients with muscle-invasive bladder cancer. The majority of studies evaluated suggested that treatment delay resulted in worse prognosis and higher pathologic stage, and the authors suggest that treatment should be performed within 12 weeks of diagnosis to ensure there is no harm to the patient (11). Similarly, Zeng et al. showed that a delay in nephrectomy for greater than 10 weeks in patients with pT3 renal cell carcinoma is associated with decreased 5-year overall survival (10). Finally, it has been well established that delay in relief of ureteral obstruction can lead to long-term renal damage (13). It is therefore not surprising that oncologic cases and endourologic cases increased proportionately within our case volume during the COVID time periods.

This study was performed in the context of previous research suggesting that our pre-operative COVID-19 screening protocol provides a safe environment for perioperative staff. Virginia Mason Medical Center is a 336 bed private hospital based in Seattle, WA with 11 full time Urologic surgeons. Currently, there is no pediatric or obstetric care at our hospital. Over the course of 5 weeks the Virginia Mason operating room performed 837 procedures on asymptomatic patients without a documented case of COVID-19 amongst procedural staff. Staff were tested if symptomatic, per institutional protocols. Prior to institution of screening, one peri-operative staff member tested positive for COVID-19.

Although the proportion of FPMRS cases decreased from 19.0% during the pre-screening period to 7.1% during the screening, the actual number of cases per week remained stable between these two time periods at 0.3 cases/day. The most common procedures performed were mesh excision followed by stage II Interstim. Options for Interstim were to cut the externalized wires at the skin and wait until the pandemic had passed or to complete the second stage. No stage I Interstim cases were initiated. Alternatives for the mesh exposure and erosion patients were to provide supportive care through reassurance, counseling, analgesia and antibiotics as necessary. However, the limitation for these options in patients who were stressed or in pain was that the temporal end point was unknown at the time of decision-making. Of note, a single discretionary sling procedure was performed. It represents a “systems error,” and emphasizes the practical difficulty of instituting hospital-wide policy change.

Transplant surgery presents a unique challenge. Suspension of renal transplant led to the loss of viable organs for patients on dialysis in a system that already has a significant shortage of organ donors (12). However, continuing transplantation needs to be considered in the setting of the immunosuppression required for allogeneic organ transplant. Admission of immunosuppressed patients to the hospital during this pandemic increase their potential exposure and susceptibility to infection and transplant recipients admitted for COVID-19 infection have a mortality rate approaching 25% (17). The American Society of Transplantation has stated that both donors and recipients should be screened, and all organ procurement should be done locally if possible (18). In an attempt to mitigate risk to living transplant donors and recipients, it was our policy to suspend living organ donation between 3/19/20-5/6/20, but to continue our deceased donor transplant program. Deceased donor recipients were also required to be local and to be confirmed not to have been sensitized. The observed trend toward fewer organ transplants during the COVID-19 pandemic compared to baseline was likely secondary to national guidelines, institutional policy, organ availability, patient preference.

The implementation of pre-operative screening during the directive to delay all elective procedures has allowed us to better care for our community. We acknowledge that operating rooms are a major driver of hospital revenue, and the financial impact of cessation of elective surgeries on hospitals has been profound. However, safety and mitigation of harm to our patients must be the primary driver behind this intervention. Deciding which patients would suffer harm through significant delay in surgical care to the extent that they require treatment in the setting of a pandemic is not a trivial decision. Using a five-tiered triage system and a multidisciplinary hospital committee to evaluate the need for every case to proceed ensured that patient well-being was the primary goal.

This 5-tiered triage system reflects the approach undertaken by other organizations (19). Rather than a tiered system, the European Association of Urology released a list of suggested procedures during this time (20). Carneiro et al. have released guideline proposals for urologic care during the COVID-19 pandemic in low- and middle-income countries. Similar to other literature, they recommend COVID-19 pre-screening when accessible, and a triaging system with continued surgical management of high-acuity issues such as >T1a renal neoplasms and bladder neoplasms (21). This approach limits unnecessary surgery during the COVID-19 pandemic. Although case volumes did increase in this study, expansion was done with the clear objective of performing procedures to manage acute disease processes, prevent harm to our patients, limit COVID-19 exposure, and conserve PPE. As the PPE shortage is relieved and emphasis changes, this study provides a model for expansion of a Urologic practice at a time when many institutions are resuming elective surgery (22).

There are several weaknesses in this study: First, it was performed in a single institution. Second, the COVID-19 pandemic is rapidly evolving. Although screening facilitates appropriate and responsible assessment of patients prior to proceeding with care, it is unclear if the same strategy will be effective as the disease prevalence changes. Finally, our community currently has a relatively low penetrance of COVID-19. Applicability to regions with far greater burden have yet to be proven.

## CONCLUSIONS

We believe that pre-procedural COVID-19 testing is a scalable intervention that will provide a means to safely reimplement care for the Urologic community. Eventually, Urologic surgical volume will need to expand nationwide in the setting of the ongoing COVID-19 pandemic and limited PPE. Universal COVID-19 screening of pre-operative patients represents a viable means to meet the needs of our patients.

## ABBREVIATIONS

PPE: Personal protective equipment
SWT: Surgical waiting time
FPMRS: Female pelvic medicine and reconstructive surgery
